# Statin therapy and recurrent venous thromboembolism in the elderly: a prospective cohort study

**DOI:** 10.1038/s41598-019-51374-8

**Published:** 2019-10-15

**Authors:** Regula Monika Kronenberg, Shanthi Beglinger, Odile Stalder, Marie Méan, Andreas Limacher, Jürg Hans Beer, Drahomir Aujesky, Nicolas Rodondi, Martin Feller

**Affiliations:** 1 Department of General Internal Medicine, Inselspital, Bern University Hospital, University of Bern, Bern, Switzerland; 2 Institute of Primary Health Care (BIHAM), University of Bern, Bern, Switzerland; 3 CTU Bern, and Institute of Social and Preventive Medicine (ISPM), University of Bern, Bern, Switzerland; 4 Department of Medicine, Division of Internal Medicine, Lausanne University Hospital, Lausanne, Switzerland; 5 Department of Internal Medicine, Cantonal Hospital of Baden, Baden, Switzerland; 6 Department of Internal Medicine, Cantonal Hospital of Luzern, Wolhusen, Switzerland

**Keywords:** Dyslipidaemias, Thromboembolism, Chronic inflammation

## Abstract

Previous studies reported lower rates of recurrent venous thromboembolism (rVTE) among statin users, but this association could be influenced by concurrent anticoagulation and confounding by statin indication. This study aimed to confirm the beneficial association between statins and rVTE, stratified according to periods with and without anticoagulation, and additionally employ propensity score weighted approach to reduce risk of confounding by indication. The setting was a prospective multicentre cohort study and the outcome was time to first rVTE in statin vs. non-statin users. 980 participants with acute VTE were enrolled (mean age 75.0 years, 47% women), with median follow-up of 2.5 years. Of 241 (24.3%) statin users, 21 (8.7%) suffered rVTE vs. 99 (13.4%) among 739 non-users. The overall adjusted sub-hazard ratio (aSHR) for rVTE comparing statin users to non-users was 0.72 (95%CI 0.44 to 1.19, p = 0.20). This association was only apparent during periods without anticoagulation (aSHR 0.50, 95%CI 0.27 to 0.92, p = 0.03; vs. with anticoagulation: aSHR 1.34, 95%CI 0.54 to 3.35, p = 0.53). Using propensity scores, the rVTE risk during periods without anticoagulation fell further (aSHR 0.20, 95%CI 0.08 to 0.49, p < 0.001). In conclusion, statin use is associated with a more pronounced risk reduction for rVTE than previously estimated, but only during periods without anticoagulation.

## Introduction

A recent systematic review and meta-analysis of randomised clinical trials (RCT) and observational studies demonstrated a 15–25% risk reduction in a first episode of venous thromboembolism (VTE) with statin use^1^. The underlying mechanism of action for this benefit could lie in a reduction of markers of inflammation^2^, resulting in a decreased coagulant state and ultimately a reduced risk for VTE^3,4^. For persons who have already suffered a VTE, an RCT with statins is lacking. According to a meta-analysis of eight observational studies, the risk of recurrent VTE (rVTE) was 27% lower under statin therapy, and this association was homogenous across the included studies (I^2^ = 0%), but results were not stratified according to whether or not patients took anticoagulants^5^.

Statin users are more likely to be older (>60 years) and suffer from cardiovascular comorbidities which predispose for VTE, compared to non-users^6–8^. In this instance, a traditional multivariable statistical analysis approach may fall short of balancing comorbidities between statin users and non-users, and the results could be confounded by statin indication^9^. Thus, in the absence of an RCT, it is advised to analyse observational data with a statistical approach that improves balance of baseline comorbidities, and this could be achieved with a propensity score weighted approach^9^.

We aimed to first assess whether statin use was associated with a lower rate of rVTE in a prospective cohort study with standardised assessment of rVTE, second to examine if the magnitude of the association between statin use and rVTE using a traditional multivariate statistical analysis approach was comparable with a propensity score (PS) weighted approach, and third to explore whether markers of inflammation (ultrasensitive C-Reactive Protein [uCRP], fibrinogen) explained the effect of statins.

## Methods

We based our study report on the guidelines provided in the Strengthening the Reporting of Observational studies in Epidemiology (STROBE) statement^10^.

### Study design, setting, and participants

This study was conducted between September 2^nd^, 2009 and December 6^th^, 2013. It was part of the Swiss Cohort of Elderly Patients with VTE (SWITCO65+) prospective multicentre cohort study, which assessed long-term medical outcomes in elderly patients with acute VTE. The study population was recruited from all five university (Basel, Bern, Geneva, Lausanne and Zurich) and four high-volume non-university hospitals (cantonal hospitals of Baden, Frauenfeld, Luzern and St. Gallen) in Switzerland. The Ethics Committee at each of the nine participating centres approved the study. All research in this observational study was performed in accordance with relevant ethics and research guidelines/regulations. Informed consent to participate in the SWITCO65+ study was obtained for all enrolled adults. Participants aged 65 years or older, with an acute, objectively confirmed symptomatic deep vein thrombosis (DVT) or symptomatic pulmonary embolism (PE), were prospectively recruited in the inpatient and outpatient settings of all study sites, and followed-up until December 6th, 2013 (participants were censored earlier in case of death or rVTE)^11^. Symptomatic PE was defined as a positive spiral computed tomography or pulmonary angiography, a high probability ventilation-perfusion scan, or proximal DVT confirmed by compression ultrasonography or contrast venography, in participants with acute chest pain, new or worsening dyspnoea, haemoptysis, or syncope. Those with catheter-related thrombosis, insufficient German-speaking or French-speaking ability, no follow-up possibility (e.g. terminal illness), an inability to provide informed consent (e.g. severe dementia), or previous enrolment in the cohort, were excluded. The SWITCO65+ study was funded by grants from the Swiss National Science Foundation (grants 33CSCO-122659/139470).

### Definition of exposures, outcomes, and potential confounders

The main exposure was statin use at study entry (statin user vs. non-user). The main outcome was the time to the first recurrence of a symptomatic, objectively confirmed VTE (rVTE) during the follow-up period, defined as PE or DVT (proximal and/or distal) based on previously published criteria^11–13^. Follow-up started directly after diagnosis of index VTE. The first follow-up took place after three months. During follow-up, study nurses interviewed participants (or respectively general practitioners in case of death, or relatives in case of no information [if participant died at home]) about the date and type of clinical events (rVTE; death). These interviews were implemented as one telephone call and two surveillance face-to-face evaluations during the first year of study participation. Following this, semi-annual contact was initiated which alternated between face-to-face evaluations (clinic visits or home visits in housebound participants), telephone calls, and periodic review of the participant’s hospital chart. If a clinical event did occur, the participant’s medical chart was reviewed, and interviews were held with the participant’s primary care physicians and family members.

In addition, information about the following variables that were relevant for the statistical analyses was collected: age (analysed as a continuous variable), smoking history (never, past, current), polypharmacy (<5 drugs vs. ≥5 drugs), prior VTE (i.e. a VTE that occurred before the index VTE), transient provoked VTE (surgery during the last 3 months, immobilisation during the last 3 months, oestrogen therapy during the last 3 months), cardiovascular disease (coronary heart disease, peripheral artery disease, cerebrovascular disease [stroke, TIA]), arterial hypertension, diabetes mellitus, body-mass index (BMI) ≥ 25 [yes vs. no], fibrinogen and uCRP (uCRP was measured at University Hospital Zurich in serum aliquots using a latex-enhanced immunoturbidimetric assay on a cobas c502 autoanalyser [Roche Diagnostics, Mannheim, Germany] with assay characteristics as reported by the manufacturer). A committee of three blinded clinical experts adjudicated all outcomes, and final classifications were made based on the full consensus of this committee^11^.

### Statistical analyses

We compared baseline characteristics among statin users and non-users with the chi-squared test and the non-parametric Wilcoxon rank-sum test as appropriate. rVTE rates were displayed for statin users and non-users, as well as stratified into periods with and without anticoagulation. We examined the overall association between statin use and the time to a first rVTE using competing risk regression, accounting for non-PE-related death as a competing event. The method yields subhazard ratios (SHR) with corresponding 95%CIs and p-values for the failure event of primary interest. In the traditional statistical analysis approach, we adjusted for established risk factors for VTE: age, sex, symptomatic PE, prior VTE, transient provoked VTE, cardiovascular disease (i.e. coronary heart, peripheral arterial or cerebrovascular [stroke, TIA] disease), active cancer, and periods of anticoagulation as a time-varying covariate. In a sensitivity analysis, we additionally adjusted for Aspirin, other antiplatelet therapy, hypercholesterolaemia and diabetes after observing that these characteristics were not evenly distributed among statin users and non-users. In another sensitivity analysis, we stratified the results into periods with and without anticoagulation. Moreover, since the study population included both provoked and unprovoked index VTE, we also performed subgroup analysis to examine whether the association between statin use and rVTE rates was similar between these two groups. Furthermore, we assessed fibrinogen and uCRP as potential inflammatory mediators, additionally correcting models for their log-transformed values. Beforehand, we performed multiple imputation for missing values of fibrinogen and uCRP.

In the PS weighted analysis approach, we calculated the propensity to receive a statin in a logistic model with statin use as dependent and all the variables that were used for adjustment in the main analysis as independent variables. The advantage of the PS approach was the ability to not only better balance the two groups (statin users and non-users) in terms of baseline characteristics, but to also account for more potential confounders if rVTE events were few. Therefore, in the PS approach we additionally adjusted for hypertension, polypharmacy, smoking status, and BMI. Missing values for BMI and smoking status were assumed to be normal (i.e. BMI as <25 [n = 4], smoking status as never [n = 3]). For completion, we compared the results from the PS approach with a sensitivity analysis from the traditional statistical approach, which adjusted for all the same variables as in the PS analysis.

We trimmed the PS values using the 2.5^th^ percentile of the PS in the statin users as the lower cut-off and the 97.5^th^ percentile of the PS in the non-users as the upper cut-off. Observations in both groups that had PS values beyond these two cut-offs were dropped^14^. We then calculated the inverse probability of treatment weights (IPTW) for the remaining participants. The competing risk model was weighted according to the IPTW and additionally adjusted for periods of anticoagulation as a time-varying covariate. Again, we stratified the results into periods with and without anticoagulation in sensitivity analyses, and also assessed fibrinogen and uCRP as potential inflammatory mediators, additionally correcting models for their log-transformed values. We used Stata 15 for all analyses (Stata Corporation, College Station, Texas).

### Essentials

- Statin users have lower rates of recurrent venous thromboembolism (rVTE), but the magnitude of this association is uncertain and could be influenced by concurrent anticoagulation.
- While statin therapy was not associated with reduced risk of rVTE during periods of anticoagulation, we observed a 50% reduced risk of rVTE with statin therapy during periods without anticoagulation.
- Using a propensity score weighted approach, statins were associated with an 80% reduced risk of rVTE during periods without anticoagulation.

## Results

### Participants

**Figure 1:**
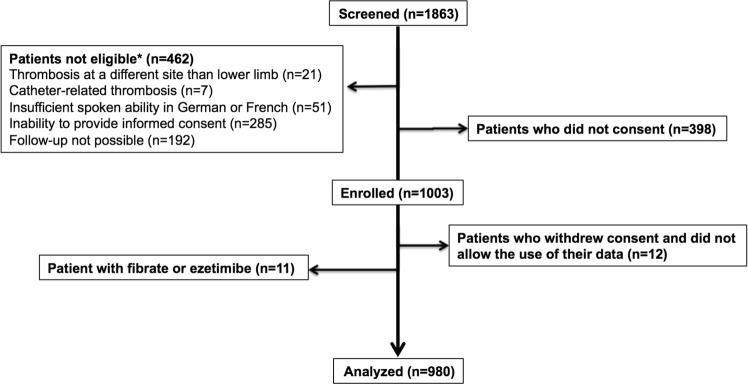
Flow-chart enrolment process.

Between September 2009 and March 2012, 1,003 participants with VTE were enrolled and followed until December 2013^11^. For this analysis, we excluded 11 participants with fibrate or ezetimibe treatment (Fig. 1), because these drugs are possibly associated with a higher risk of VTE^15,16^. We further excluded four participants who withdrew informed consent within one day, and eight participants who denied use of their data.

The 980 included participants had a mean age of 75.0 years, 52.7% were men, 99.8% were Caucasian. The majority of participants had comorbidities, such as active cancer, chronic lung disease, or heart failure (Table 1). Overall, 69.3% of participants had PE at baseline, with or without DVT. Most participants (81.2%) were treated in hospital, and low-molecular-weight heparin was administered to 48.6% of participants as initial antithrombotic treatment.

**Table 1:** Baseline characteristics of statin users and non-users^a^.

Total N	Statin	No statin	p-value
	n (%) or median (IQ-range)		
	N = 241	N = 739	
Age [years]	75.0 (70.0; 81.0)	75.0 (69.0; 81.0)	0.63
Females	107 (44%)	351 (47%)	0.40
Smoking status			0.77
current smoker	18 (7%)	58 (8%)	
past smoker	103 (43%)	295 (40%)	
never smoker	120 (50%)	383 (52%)	
Family history of pulmonary embolism/deep vein thrombosis	37 (15%)	125 (17%)	0.57
Known thrombophilia	2 (1%)	11 (1%)	0.44
Diabetes mellitus	61 (25%)	91 (12%)	<0.01
Hypercholesterolaemia	206 (85%)	87 (12%)	<0.01
Coronary heart disease	101 (42%)	68 (9%)	<0.01
Peripheral arterial disease	29 (12%)	40 (5%)	<0.01
Cerebrovascular disease (stroke/ transient ischaemic attack)	50 (21%)	40 (5%)	<0.01
Cardiovascular disease (coronary/peripheral/			
cerebrovascular)	180 (75%)	148 (20%)	<0.01
Arterial hypertension			<0.01
no	49 (20%)	301 (41%)	
yes (treated)	189 (78%)	416 (56%)	
yes (untreated)	3 (1%)	22 (3%)	
Chronic lung disease	34 (14%)	102 (14%)	0.91
Body-mass index [kg/m^2^]	27.7 (24.6; 30.8)	26.4 (23.8; 29.7)	<0.01
Body-mass index ≥25	174 (72%)	463 (63%)	<0.01
Symptomatic pulmonary embolism	186 (77%)	491 (66%)	<0.01
Prior venous thromboembolism^b^	75 (31%)	203 (27%)	0.28
Major surgery during the last 3 months	44 (18%)	102 (14%)	0.09
Current oestrogen therapy during the last 3 months	4 (2%)	28 (4%)	0.11
Immobilisation during the last 3 months	57 (24%)	159 (22%)	0.49
Provoked venous thromboembolism	75 (31%)	219 (30%)	0.66
Active cancer	38 (16%)	138 (19%)	0.31
History of major bleeding	37 (15%)	63 (9%)	<0.01
Chronic renal disease	67 (28%)	117 (16%)	<0.01
Polypharmacy	193 (80%)	304 (41%)	<0.01
Anticoagulation prior to venous thromboembolism	19 (8%)	32 (4%)	0.03
Initial parenteral anticoagulation	232 (96%)	714 (97%)	0.80
Type of initial parenteral anticoagulation			0.02
Low molecular weight heparin	94 (39%)	366 (50%)	
Unfractionated heparin	97 (40%)	233 (32%)	
Fondaparinux	40 (17%)	115 (16%)	
Danaparoid	1 (0%)	0 (0%)	
None	9 (4%)	25 (3%)	
Initial vitamin K antagonist therapy	211 (88%)	640 (87%)	0.705
Concomitant antiplatelet therapy	149 (62%)	166 (22%)	<0.001
Aspirin	127 (53%)	154 (21%)	<0.001
Duration of initial anticoagulation [months]	11.1 (5.2; 28.7)	7.1 (3.7; 23.7)	0.012
Fibrinogen at the time of venous thromboembolism [g/L]	4.8 (3.7; 5.8)	4.5 (3.6; 5.6)	0.102
Ultrasensitive C-Reactive Protein at the time of venous thromboembolism [mg/L]	22.2 (6.1; 63.6)	23.6 (8.5; 62.1)	0.260

^a^Missing: 3 (0%) for smoking status, 8 (1%) for family history of venous thromboembolism, 18 (2%) for systolic blood pressure, 4 (0%) for body-mass index, 1 (0%) for oestrogen therapy, 1 (0%) for history of major bleeding, 125 (13%) for fibrinogen, 124 (13%) for ultrasensitive C-Reactive Protein.

^b^Venous thromboembolism prior to the index venous thromboembolism.

241 (24.3%) participants reported statin use compared to 739 non-users. Among the statin users, 103 took atorvastatin, 73 simvastatin, 49 pravastatin, 12 rosuvastatin, and 4 fluvastatin. Statin users and non-users had a similar age and sex distribution, but statin users were more overweight (BMI ≥ 25 kg/m^2^: 72% vs. 62%) and were generally in a worse medical condition suffering from more comorbidities, with a higher prevalence of diabetes mellitus (25% vs. 12%), arterial hypertension (79% vs. 59%), chronic or acute heart failure (19% vs. 9%), coronary heart disease (42% vs. 9%), peripheral arterial disease (12% vs. 5%), cerebrovascular disease (21 vs. 5%), past major bleeds (15 vs. 9%), and polypharmacy (80 vs. 41%).

### Statins and rVTE

After a mean follow-up of 27.5 months (median 29.6 months), 21 of the 241 statin users (8.7%) experienced a rVTE compared to 99 of the 739 non-users (13.4%). rVTE incidence rates (IR) were 3.8 per 100 patient-years (95%CI 2.5 to 5.9) among statin users vs. 6.3 per 100 patient-years (95%CI 5.2 to 7.7, p = 0.036) among non-users. During periods with anticoagulation, rVTE IR were 2.5 per 100 patient-years (95%CI 1.3 to 4.8) among statin users vs. 2.3 per 100 patient-years (95%CI 1.5 to 3.5) among non-users. During periods without anticoagulation, rVTE IR were 6.4 per 100 patient-years (95%CI 3.6 to 11.3) among statin users vs. 12.6 per 100 patient-years (95%CI 10.1 to 15.8) among non-users. Overall, statin users had a 38% reduced risk of rVTE during follow-up compared to non-users (crude SHR 0.62, 95%CI 0.39 to 1.00, p = 0.05). After adjusting for age, sex, symptomatic PE, prior VTE, transient provoked VTE, cardiovascular disease, active cancer, and periods of anticoagulation as a time-varying covariate, the effect estimate was slightly attenuated (adjusted [a]SHR 0.72, 95%CI 0.44 to 1.19, p = 0.20; Table 2). In the sensitivity analysis additionally adjusting for Aspirin, other antiplatelet therapy, hypercholesterolaemia and diabetes, statins were more strongly associated with rVTE (aSHR 0.56, 95%CI 0.32 to 0.99, p = 0.05). When only time-periods with anticoagulation were considered, there was no association between statin use and rVTE (aSHR 1.34, 95%CI 0.54 to 3.35, p = 0.53). In contrast, when only time-periods without anticoagulation were considered, statin use was more strongly associated with rVTE (aSHR 0.50, 95%CI 0.27 to 0.92, p = 0.03). Upon subgroup analysis, the incidence rates of rVTE were slightly lower in patients with a provoked index VTE (IR 4.6 per 100 patient-years, 95%CI 3.2 to 6.6) compared to unprovoked index VTE patients (IR 6.1 per 100 patient-years, 95%CI 5.0 to 7.5). Nevertheless, reduction in rVTE risk with statin use compared to non-use was still similar among the two subgroups (provoked index VTE - aSHR 0.71, 95%CI 0.24 to 2.15, p = 0.55; unprovoked VTE - aSHR 0.68, 95%CI 0.39 to 1.20, p = 0.19); confirming that there was no observable subgroup effect (p for interaction 0.93). Neither fibrinogen nor uCRP had an impact on the results (aSHR including fibrinogen 0.75, 95%CI 0.45 to 1.23, p = 0.25; aSHR including uCRP 0.72, 95%CI 0.43 to 1.19, p = 0.20; Table 2).

**Table 2:** Statin therapy and rVTE.

Number of events/Number of patients	Analysis	Crude subhazard ratio (95%-CI)	p-value	Adjusted subhazard ratio (95%-CI)	p-value
	**Traditional statistical analysis approach**				
120/980	**Overall**	0.62 (0.39 to 1.00)	0.049	0.72 (0.44 to 1.19)^a^	0.197
31/972	**Periods with AC**	1.16 (0.54 to 2.49)	0.707	1.34 (0.54 to 3.35)^a^	0.525
89/595	**Periods without AC**	0.52 (0.28 to 0.95)	0.034	0.50 (0.27 to 0.92)^a^	0.027
	Sensitivity analysis including same variables as the propensity score approach			0.60 (0.36 to 1.01)^b^	0.056
	Including markers of inflammation				
120/980	Fibrinogen			0.75 (0.45 to 1.23)^c^	0.250
120/980	ultrasensitive CRP			0.72 (0.43 to 1.19)^c^	0.197
	**Propensity score weighted approach** ^**d**^				
99/792	**Overall**	0.60 (0.34 to 1.03)	0.064	0.42 (0.21 to 0.81)^e^	0.010
28/788	**Periods with AC**	1.34 (0.60 to 3.01)	0.481	1.17 (0.47 to 2.90)^e^	0.740
71/477	**Periods without AC**	0.38 (0.18 to 0.84)	0.016	0.20 (0.08 to 0.49)^e^	<0.001
	Including markers of inflammation				
99/792	Fibrinogen			0.43 (0.22 to 0.84)^c^	0.014
99/792	ultrasensitive CRP			0.41 (0.21 to 0.80)^c^	0.009

AC = anticoagulation, CRP = C-Reactive Protein.

^a^Adjusted for age, gender, symptomatic pulmonary embolism, prior venous thromboembolism, provoked venous thromboembolism, cardiovascular disease (i.e. coronary heart, peripheral arterial or cerebrovascular [stroke, transient ischaemic attack] disease), active cancer, and periods of anticoagulation as a time-varying covariate.

^b^Sensitivity analysis: further adjustment for the same variables as used in the propensity score approach. Adjusted to include age, gender, symptomatic pulmonary embolism, prior venous thromboembolism, provoked venous thromboembolism, cardiovascular disease (i.e. coronary heart, peripheral arterial or cerebrovascular [stroke, transient ischaemic attack] disease), active cancer, periods of anticoagulation as a time-varying covariate, and additionally hypertension, polypharmacy, smoking (never/past/current), body-mass index (≥25).

^c^Two separate additional adjustment variables: log-Fibrinogen and log-ultrasensitive C-Reactive Protein (as potential explanatory variables of the association between statins and rVTE). Multiple imputation for missing values of fibrinogen and ultrasensitive C-Reactive Protein was performed.

^d^Variables used to calculate the propensity score: age, gender, symptomatic pulmonary embolism, prior venous thromboembolism, provoked venous thromboembolism, cardiovascular disease (i.e. coronary heart, peripheral arterial or cerebrovascular [stroke, transient ischaemic attack] disease), active cancer, hypertension, polypharmacy, smoking (never/past/current), and body-mass index (≥25).

^e^The adjusted model was weighted according to inverse probability of treatment weights (IPTW) and additionally adjusted for periods of anticoagulation as a time-varying covariate.

In the PS weighted analysis (based on 792 participants with 99 rVTEs), we observed a more pronounced rVTE risk reduction of 58% among statin users compared to non-users (aSHR 0.42, 95%CI 0.21 to 0.81, p = 0.01). Again, the association became stronger during periods without anticoagulation (aSHR 0.20, 95%CI 0.08 to 0.49, p < 0.001), whereas it was not observed during periods of anticoagulation (aSHR 1.17, 95%CI 0.47 to 2.90, p = 0.74). Further, neither fibrinogen nor uCRP had an impact on the results (aSHR including fibrinogen 0.43, 95%CI 0.22 to 0.84, p = 0.01; aSHR including uCRP 0.41, 95%CI 0.21 to 0.80, p = 0.01; Table 2). In a sensitivity analysis using the traditional statistical analysis approach, but adjusting for all variables that were used in the PS analysis, the association between statin use and rVTE was slightly stronger compared to the original multivariable approach (aSHR 0.60, 95%CI 0.36 to 1.01, p = 0.06 vs. aSHR 0.72, 95%CI 0.44 to 1.19, p = 0.20).

## Discussion

In this population-based prospective cohort study, we found a lower rate of rVTE among statin users compared to non-users, but this beneficial association was only apparent during periods without anticoagulation, and stronger in the PS weighted approach than in the traditional statistical analysis approach. Furthermore, the potentially beneficial effect of statins was not explained by markers of inflammation such as fibrinogen or uCRP.

Our results from the traditional statistical analysis approach were very similar to a recent meta-analysis of eight observational studies^5^, indicating an almost 30% risk reduction in rVTE with statin therapy. However, our results suggest that statins may only reduce the risk for rVTE in the absence of anticoagulation. Therefore, they might be a second best option to prevent rVTE when anticoagulation is contraindicated. Moreover, under the PS weighted approach, the risk reduction became even more substantial (i.e. 80% during periods without anticoagulation). There are arguments supporting the hypothesis that the traditional statistical analysis approach underestimates the true effect of statins in observational studies: statin users have more comorbidities and are sicker than non-users^6,7^, which is also observed in our own study population’s baseline characteristics (Table 1), and multimorbid persons have a higher risk of VTE^8^. Thus, the potentially beneficial effect of statins could be partly concealed by comparing multimorbid statin users with healthier non-users in observational studies using a traditional multivariable statistical analysis approach. In contrast, in the PS weighted approach, participants with a PS value below the 2.5^th^ percentile and above the 97.5^th^ percentile were discarded, as at these extremes of range there was no comparable participant in the other group with similar comorbidities/risk profile, thus making the two groups (statin users vs. non-users) as similar as possible^14^.

Our study has several limitations. First, the study population was recruited consecutively from most large-volume hospitals in Switzerland. As a result, it is uncertain if the results also apply to patients with potentially less severe (r)VTE who were treated in smaller hospitals or by general practitioners. Second, almost all study participants were white, so our results are only generalisable to a Caucasian population. Third, we tested if the potential beneficial effect of statins was explained by a reduction in inflammation – we observed no such association. Yet, we only considered fibrinogen and uCRP. Both markers have been suggested by previous studies to be affected by statin therapy^17,18^. It is possible that statins reduce the risk of (r)VTE via other inflammatory markers for which we had no information available or via direct impact on endothelial function^17,19^. In fact, newly published data suggests that the protective effect of statins in decreasing rVTE rates may not be down to a reduction in inflammation, but rather due to a reduction in coagulation factors^20^. Fourth, our study did not have sufficient power to analyse different statin agents separately, and it is possible that the risk reduction seen in (r)VTE is statin-specific and therefore, cannot be attributed to a class-effect^21,22^. Fifth, all participants were 65 years or older. Therefore, it is uncertain whether our results also apply to a younger population. Sixth, whether or not a participant used statins was only assessed at baseline. Therefore, our exposure assessment (i.e. statin use) might be inaccurate in cases where participants started or stopped statin therapy during follow-up. However, as this inaccuracy is not related to the outcome of interest (rVTE), results may at worst be biased towards the null (i.e. underestimate the association between statin use and rVTE).

Our study has several strengths. First, it highlights that statistical analysis approaches accounting for confounding by indication (i.e. the PS weighted approach) can produce different results from traditional multivariable analyses of observational data. Analysing observational data with both a traditional multivariable analysis and a PS weighted analysis, comparing the findings and attempting to explain differences, can result in new insights. Furthermore, it makes optimal use of already existing data. Second, our study included elderly patients with VTE (mean age 75 years), a population that is often underrepresented or excluded from clinical trials. Third, the cohort had a small dropout rate of 4.8% during follow-up^11^, and the data collection was near complete with less than 5% missing values. Fourth, rVTE was adjudicated by a blinded expert committee using clearly defined previously published objective criteria^11–13^, reducing the risk of detection bias. Finally, both inpatients and outpatients with an index VTE were included in a nationwide multicentre setting.

## Conclusion

Statin use was associated with a more pronounced risk reduction for rVTE than previously estimated, but only without concurrent anticoagulation. An RCT would be needed to substantiate the potential benefits of statins for prevention of rVTE during periods when anticoagulation is not indicated or feasible.

## Data Availability

All necessary data that support the findings of this study are included within this published article. Any further supplemental data is available from the authors upon request.
